# Ethosomal Gel Formulation of Alpha Phellandrene for the Transdermal Delivery in Gout

**DOI:** 10.34172/apb.2021.015

**Published:** 2020-11-07

**Authors:** Sony Valsalan Soba, Merin Babu, Rajitha Panonnummal

**Affiliations:** ^1^Amrita School of Pharmacy, Amrita Institute of Medical Science & Research Center, Amrita VishwaVidyapeetham, Kochi-682041 India.

**Keywords:** Alpha phellandrene, Terpene, Ethosomal gel, Anti-inflammatory, Gout

## Abstract

***Purpose:*** Purpose was to improve the skin compatibility and permeability of alpha phellandrene through an ethosomal gel formulation for the treatment of gout; as the oral use of the drug is reported to cause gastrointestinal disturbances and toxicities.

***Methods:*** Alpha phellandrene loaded ethosomal formulation (APES) was prepared by cold method for the treatment of gout. APES were loaded into carbopol gel (APEG) by dispersion method. Physico-chemical characterizations of the APES were done by dynamic light scattering (DLS), transmission electron microscopy (TEM), Fourier-transform infrared spectroscopy (FTIR) etc. *In vitro* release, permeation, haemo-compatibility and anti-inflammatory studies were conducted.

***Results:*** APES showed a particle size of 364.83 ± 45.84 nm. The entrapment efficiency of the optimized formulation is found as 95.06 ± 2.51%. Hemolysis data indicated that APES does not cause any significant hemolysis. *In vitro* drug release studies were carried out using dialysis membrane technique and the amount of drug released from APES & APEG is found to be 95% and 94.21% respectively after 5 and6 hours. Kinetic data analysis revealed that APES & APEG follows first order and zero order release kinetics, respectively. The anti-inflammatory activity studies of the formulation are done by estimating its inhibitory effects on cyclooxygenase II (COX) II, lipoxygenase-5 (LOX-5), Myeloperoxidase (MPO), Inducible nitric oxide synthase (INOS) & cellular nitrite level using RAW 264.7 cells. The significant inhibition in the activities of the enzymes implies the anti-inflammatory activity of the formulations. Skin permeation study was carried out using porcine skin and revealed that the permeation of alpha phellandrene is increased from APES & APEG when compared with alpha-phellandrene solution (APS). Skin deposition study of APS, APES & APEG revealed better drug deposition from APEG (48.799 ± 1.547µg/cm^2^ ) after 24 hours when compared with APS & APES.

***Conclusion:*** Overall results indicate that the ethosomal formulation of alpha phellandrene through transdermal route is an effective alternative for oral use of the drug.

## Introduction

Gout is a condition characterized by increased level of uric acid in blood (hyperuricemia) and it is deposited in various tissues as monosodium urate (MSU) crystals. MSU crystals are deposited in all tissues, mainly in & around the joints leads to the formation of tophi. Gout is associated with other metabolic disorders such as obesity, diabetes mellitus, cardiovascular problems & the prevalence rate of gout in adults is reported as 1% to 2%.^1,2^ Apart of many hereditary disorders associated with excretion of uric acid & metabolism of purine; the major cause of gout is considered to be the consumption of uric acids from diet, often in the form of purines.^3^ Arthritis associated with gout is the most common form of arthritis & painful arthritic attack occur at overnight. Prevalence rate of gouty arthritis is 1.4% and it rises with age.^4^ Acute gout appears when the urate crystals are deposited immediately in joints cause gouty inflammation & intense pain. The immediate attack is referred to as a “flare” & will normally weaken within 3 -10 days. Drugs which are used for treatment of acute gout are non-steroidal anti-inflammatory drugs (NSAIDs), colchicine and steroids.^5^ In chronic gout, gouty attack increase continuously and leads to joint damage with chronic pain. Here the MSU developed as large stones and deposited in pinna of the external ear, eyelids, nose & around the joints. Deposition of urate crystals in kidney leads to renal disease. Drugs available for the treatments of chronic gout are allopurinol, probenecid and sulfinpyrazone etc.^6^

Gout is a major problem worldwide and significant advances in the treatment of gout have been made in past few decades. The current available treatment options are not completely satisfactory and are associated with so many side effects. In addition, due to economic problem in developing countries like India, even today modern health care is out of reach for most of the population.

The use of bioactive natural products for the treatment of various ailments is increasing nowadays as they are considered as much safer when compound with synthetic drugs. The various medicinal plants are used in the treatment of gout. These plants exhibit anti-gout effect by different mechanisms, such as xanthine oxidase inhibition, uricosuric activity, anti-inflammatory activity and anti-oxidant activity.^7,8^

Alpha phellandrene is a terpene isolated from the root of *Moringa oleifera.* Alpha phellandrene is a cyclic monoterpene, that can occur as enantiomeric forms (- and + isomers) and is present in volatile oil of several plants. Alpha phellandrene exerts anti-inflammatory action by different mechanisms such as by inhibiting neutrophil migration towards the site of inflammation & by stabilizing of mast cell (there by prevent the release of inflammatory mediators). Alpha phellandrene is reported to have the potential to use for the treatment of inflammatory diseases, like rheumatoid arthritis, osteoarthritis, allergic disease etc.

Adverse skin reactions are reported on topical application of alpha phellandrene. As per report when undiluted solution of alpha phellandrene is applied to rabbit skin moderate skin irritation occurs. Oral ingestion of alpha phellandrene is reported to cause nausea, vomiting, diarrhea and intestinal disturbances.

Ethosomes are non-invasive drug delivery carriers that enable drugs to reach into the deep layer of skin and/ or the systemic circulation. The high concentration of ethanol makes the ethosome unique, as ethanol disrupt the skin lipid bilayer organization and there by improves the vesicles ability to penetrate into the stratum corneum. Also, because of the high ethanol concentration, the lipid membrane is packed less tightly than conventional vesicles but with equivalent stability, allowing a more malleable structure and improves the drug distribution ability in stratum corneum lipids.^9-11^ The ethosomal vesicles size is tens of nanometers to microns.^12^

So the present work aims to overcome the demerits of oral alpha phellandrene and to reduce the skin irritation potential of topical alpha phellandrene through an ethosomal gel formulation for the treatment of gout.

## Materials and Methods

### Materials

Materials used for the study were purchased from different companies and are shown in Table 1.

**Table 1:** List of companies from which all materials needed for the study have been purchased

**Name of material**	**Specification**
Alpha phellandrene	Sigma Aldrich
Soya lecithin	Himedia laboratories Pvt. Ltd. Mumbai
Cholesterol	Nice Chemicals
Propylene glycol	LOBA Chemie Pvt. Ltd
Ethanol	Nice Chemicals
Sodium Chloride	Nice Chemicals
Triton X-100	LOBA Chemie Pvt. Ltd
Formaldehyde	Nice Chemicals
Potassium dihydrogen phosphate	Spectrum Reagents, Kochi
Dipotassium dihydrogen phosphate	Qualigens Fine Chemicals, Mumbai
Sodium dihydrogenphosphate	Fisher Scientific
Disodium hydrogen phosphate	Fisher Scientific
Carbopol 940	LOBA Chemie Pvt. Ltd

### Methods

#### Preformulation studies

Preformulation studies are conducted to investigate and confirm the physical and chemical properties of drug and excipients. Following studies were performed

### FTIR spectral analysis

The Fourier-transform infrared spectroscopy (FTIR) analysis of the drug was carried out by using a furrier transform IR analyzer (Made-Shimadzu Kyoto, Japan) and the spectra obtained complies with that reported for reference standard.

### Organoleptic evaluation

Organoleptic characters like color, dour and taste of alpha phellandrene were evaluated and recorded by descriptive terminology.

### Absorption maximum of the drug

Absorption maximum of alpha phellandrene was determined using ethanol as solvent. Different concentrations of drug ranging from 10-60 µg/mL were prepared and analyzed by UV spectrophotometer (Made-UV-1700, Shimadzu; Kyoto, Japan).

### Miscibility studies

The miscibility studies of alpha phellandrene was carried out using different solvents like water, ethanol, ether, dilute solution of alkali hydroxide, carbonates and mineral acids.

### Development of alpha phellandrene loaded ethosomal suspension (APES)

Ethosomes were prepared by solubilizing required amount of soya lecithin with ethanol (containing drug alpha phellandrene) by mixing using the magnetic stirrer (Remi). During the stirring process, small quantity of propylene glycol was added. The assembly was covered to avoid the evaporation of ethanol. Distilled water was added slowly with continuous stirring to assist the formation of ethosomal suspensions. The final ethosomal suspension was kept at room temperature for 30 min with continuous stirring. Quantities of various ingredients used for the preparation of alpha phellandrene loaded ethosomal formulation (APES) were shown in Table 1. The prepared ethosomal suspensions were centrifuged at 20 000 rpm for 3hr and the formulation without any phase separation is considered as stable and was taken for further tests. Formulations were stored in the refrigerator.^13^

### Preparation of alpha phellandrene loaded ethosomal gel (APEG)

Ethosomal gel can be prepared by dispersion method using carbopol 940 as polymer. Carbopol 940 was dispersed with distilled water and allow for swelling at overnight. This mixture was then neutralized by drop wise addition of triethanolamine. Glycerol was added to the gel for maintaining sufficient viscosity. To this gel solution ethosomal dispersion was added and mixed properly. Mixing was continued until transparent gel formulation was appeared. Paraben was used as preservative. Quantities of various ingredients used for the preparation of APEG were shown in Table 2. Final formation of gel was filled in a glass vials and stored at the temperature 4-8°C.^14^

**Table 2:** Organoleptic evaluation report of Alpha phellandrene

**Parameters**	**Standard**	**Observation**
Colour	Colourless to light yellow colour	Colourless to light yellow colour
Odour	Medium, terpene	Medium, terpene
Taste	Spicy	Spicy

### Characterization of alpha phellandrene loaded ethosomal suspension

Prepared APES was characterized for its particle size, shape and surface charge as follows.

### Particle size analysis & zeta potential

Particle size & size distribution of the alpha phellandrene loaded ethosomal suspension was determined by dynamic light scattering method (DLS zeta sizer) using a computerized inspection system. DLS measures the hydrodynamic diameter of particles and particle size dispersion is indicated by the polydispersity index. Zeta potential is expressed in mv.^15^

### Transmission electron microscopy

Shape and morphology of APES was examined by transmission electron microscopy (TEM) (FEI Tecnai F20 G2). For TEM analysis a drop of ethosomal suspension was diluted with distilled water, deposited on a carbon coated copper grid, stained with phosphotungstic acid (2% w/v), allowed to dry and evaluated using TEM.^16^

### Infrared spectroscopic analysis

Infrared (IR) spectroscopy is used to detect the functional groups present in a molecule, and which helps to confirm the presence or absence of functional groups present in a molecule. FTIR spectral analysis of alpha phellandrene and APES was carried out.

### Entrapment efficiency

Entrapment efficiency of APES was carried out by e indirect method. In this technique, AEPS were centrifuged at 20 000 rpm at 37°C for 3 hours. After the centrifugation, the supernatant was diluted with ethanol and the free drug concentration (alpha phellandrene) in the supernatant was determined by UV spectroscopy analysis at 263 nm.

Entrapment efficiency (%) = C_t_-C_s_/ C_t_ * 100

Ct – complete amount of medication added

Cs– amount of medication present within the supernatant.

### Quantification of alpha phellandrene

For the preparation of calibration curve a stock solution of alpha phellandrene was prepared by adding 10 mg of alpha phellandrene into the 100 ml volumetric flask and make up to the volume with ethanol so as to get a final concentration of 100 µg/mL. From this stock solution, added 0.1, 0.2, 0.3, 0.4, 0.5, 0.6 mL into 10 ml volumetric flasks and made up to the volumes to obtain the concentrations of 1 to 6 µg/mL. The absorbance was measured at 263 nm from UV spectrophotometer using ethanol as blank. The absorbance was plotted against concentrations to plot the standard graph. The linear regression equation obtained was used to calculate from the amount of methotrexate in further studies.

### In vitro hemolysis assay

Collected fresh human blood in acid-citrate-dextrose coated tubes and then it was centrifuged (Hermle Labortechnik, Germany). Collected RBCs were treated with 1 mL of various concentrations of (20, 40, 60, 80 and 100 μg/mL) of APES. All the mixtures were incubated at 37°C for 30 minutes and spinned. The content of hemoglobin in the supernatant solution was determined at 560 nm using UV.^17^ The percentage hemolysis was calculated using the following equation.

Percent of hemolysis = OD of test /OD of control * 100

### In vitro release studies and kinetic data analysis

### In vitro drug release study

The dialysis bag method was used for the study of *in vitro* release of alpha phellandrene from APES. 2 ml of formulation was introduced into open tubes whose one is tied with in the dialysis membrane. The assembly was dipped in 30 mL of phosphate buffer of pH 7.4, maintained at temperature of 37°C and was stirred with magnetic stirrer. Samples were withdrawn at pre-decided time intervals and were replaced with same amount of buffer. Sink conditions was maintained throughout the study. The amount of drug released was determined using UV spectrophotometer set at 263 nm.^18,19^ Cumulative % drug released vs time graph was plotted.

### Kinetic modelling of data

*In vitro* drug release study data was subjected to different kinetic model analysis as- zero order drug release kinetics, first order release kinetics, Higuchi model and Korsmeyer-Peppas models so as to determine the mechanism and drug release kinetics.^20,21^

### Cell line studies

Cell line studies were performed using RAW 264.7 cell line. RAW 264.7 cells were obtained from National Centre for Cell Sciences, Pune, India and cultured in Dulbecco’s improved Eagles medium, DMEM supplemented with 10% FBS, L-glutamine, sodium bicarbonate (Merck, Germany) and antibiotic solution containing: penicillin (100 U/mL), streptomycin (100 µg/mL), and amphoteracin B (2.5 µg/mL) in 25 cm^2^ tissue culture flask kept at 37ºC in a CO_2_ incubator (Esco) maintained 5% of CO_2_ .The cells were allowed to reach at 60% confluency and then activated by adding 1 µL of lipopolysaccharide (LPS: 1 µg/mL). LPS stimulated cells were exposed with different concentrations (25, 50, 100 µg/mL) of samples for 24 hours. Diclofenac sodium was used as standard for comparison of anti-inflammatory activity.

### Cyclooxygenase II (COX II) activity

Method of Walker and Gierse was used for the estimation of COX activity. The cell lysate was incubated with Tris-HCl buffer (pH 8), glutathione 5 mM/L, and haemoglobin 5 mM/L at 25°C and initiated the reaction by adding 200 mM/L of arachidonic acid. Incubated for 20 minutes at 37°C and the reaction was terminated after by the addition of 10% trichloroacetic acid in 1 N hydrochloric acid. After the centrifugal separation, added 1% thiobarbiturate and the tubes were boiled for 20 minutes. After cooling, the tubes were centrifuged for three minutes and determined the COX activity by measuring the absorbance at 632 nm.^22-24^

Percentage inhibition of the enzyme was calculated as,

% inhibition = (Absorbance of control-Absorbance of test)/Absorbance of control) × 100

### Lipoxygenase-5 (LOX-5) activity

LOX activity was measured by using the method of Axelrod. The reaction mixture (2 mL final volume) contained Tris-HCl buffer (pH 7.4), cell lysate (50 µL) and sodium linoleate (200 µL). The LOX activity was measured as an increase of absorbance at 234 nm (Shimadzu), which reflects the formation of 5-hydroxyeicosatetraenoic acid.

Percentage inhibition of the enzyme was calculated as, % inhibition = (Absorbance of control-Absorbance of test)/Absorbance of control) × 100

Method of Axelrod et al was used for LOX assay. The reaction mixture (2 mL final volume) contained Tris-HCl buffer (pH 7.4), cell lysate (50 µL), and sodium linoleate (200 µL). The LOX activity was measured as an increase of absorbance at 234 nm (Shimadzu), which reflects the formation of 5-hydroxyeicosatetraenoic acid.^12^ Percentage inhibition of the enzyme was calculated

### Myeloperoxidase (MPO) activity

The cell lysate was homogenized in a solution containing 50 mM potassium phosphate buffer and 0.57 % hexadecyl trimethyl ammonium bromide. For the determination of of myeloperoxide(MPO) activity the samples are centrifuged at 2000g and 4 0C for 30 minutes. To this added 50 mM phosphate buffer (pH 6) and 1.67 mg/mL of guaiacol in H2O; so as to activate the enzyme myeloperoxidase(MPO) The change in absorbance at 460 nm was measured. MPO activity was presented as units per mL of cell lysate. One unit of MPO activity was defined as that the enzyme needed to degrade 1 µM of peroxide per minute at 25°C.^23,24^

Enzyme activity; U = (ΔOD * 4 * Vt * dilution factor) / (L · E 470 *Δt *Vs)

ΔOD = density change

Vt = total volume (mL) (1.1 mL)

L=light path (1 cm)

E 470 = extinction coefficient for tetraguaiacol

T is the time of measurement in minutes and Vs is sample volume in mL.

### Inducible nitric oxide synthase (INOS)

Salter et al method was used to determine the nitric oxide synthase activity. The assay system contained substrate, L-Arginine (0.1 mL -2 µmol/L), manganese chloride (0.1 ml - 4 µmol/L), dithiothreitol (0.1 mL – 10 mmol/L), NADPH (0.1 mL- 1 mmol/L), tetrahydropterin (0.1 mL - 4 µmol/L) , oxygenated haemoglobin (0.1 mL 10 µmol/L) and enzyme (0.1 mL -sample). The absorbance was measured at 401 nm.^21-24^

Percentage inhibition of the enzyme was calculated as,

% inhibition = (Absorbance of control-Absorbance of test)/Absorbance of control) × 100

### Estimation of Cellular Nitrite Levels

Method of Lepoivre et al was used to determine the cellular nitrite level. To 0.5 ml of cell lysate added 0.1 mL of sulphosalicylic acid and vortexed well for 30 minutes. The samples were then centrifuged at 5000 rpm for 15 minutes. Nitrite level was determined using the protein-free supernatant. Added 30 μL of 10% NaOH to the 200 μL of the supernatant followed by 300 μL of Tris-HCl buffer and mixed well. To this, added 530 μL of Griess reagent mixture (1% sulphanilamide, 2% phosphoric acid and 0.1% N-1-naphthyl ethylene diamine dihydrochloride) and incubated in the dark for 10–15 minutes. The absorbance was measured at 540 nm against a Griess reagent as blank. Sodium nitrite solution was used as the standard. The amount of nitrite present in the samples was determined from the standard curves obtained.^22-25^

### In vitro skin permeation, deposition and histological studies

#### In vitro skin permeation study

The permeation experiments were carried out by utilizing full width of the porcine skin mounted in a vertical Franz diffusion cell. Porcine skin sample was selected because of the histological and biochemical similarity with human skin. Furthermore, the stratum corneum, epidermal and the dermal width of the porcine ear skin are related to human skin.^26^

### Preparation of Franz diffusion cell

The surface area of the Franz diffusion cell (Made-Orchid Scientifics – FDC-06) was 2.54 cm^2^ and receptor compartments with a capacity of approximately 7 mL was used for conducting permeation experiments. The receptor compartment was filled with PBS pH 7.4 and the thawed skin samples were transferred onto the Franz diffusion cell and it was sandwiched between the donor and receptor compartment in a manner that the stratum corneum side was exposed to the donor compartment and the dermal side to the receptor solution. The pre conditioning of the skin samples were done by hydrating skin samples with the phosphate buffer for 30 minutes. The temperature was set as at 32 ±2°C through a flowing water jacket and then receptor medium was continuously mixed with a magnetic bead, during the testing period.^26,27^

### Permeation experiments

The Franz diffusion cells with the porcine skin sandwiched between the donor and acceptor compartments were make ready. One milliliter of AEPS was added in to the donor compartment on the stratum corneum side of the skin. At prearranged interval of time such as 1, 2, 3, 4, 5, 6 and 24 hours, 0.5 mL of the receptor fluid were *withdrawn*   and replaced with a same amount of fresh buffer solution. The samples are observed spectrometrically at 263 nm to quantify alpha phellandrene permeated. Amount of drug penetrated was plotted against time.

### Skin deposition studies

After the skin permeation experiments the skin surface was washed three times in phosphate buffer solution. Weight of skin samples were noted after drying the samples using filter paper. Then the amount of drug deposited into the skin layers was extracted by treating of skin with specified volume of solvent ethanol. The drug content present was determined using UV Spectroscopy after centrifugation of the samples. The amount of drug deposited was expressed in microgram/ gram of skin.^28^

### Histological evaluation

After the skin permeation study, the skin samples were subjected to histological analysis. After 24 hours of study the porcine ear skin samples were fixed using 10% neutral phosphate buffered formalin. Following the fixation, samples were treated with iso-propanol, xylene and then paraffin embedded. The microsections of samples were prepared and were stained with hematoxylin and eosin. The sections were mounted using DPX and observed under optical microscope equipped with computer-controlled digital camera. The histological changes in the skin samples were evaluated with the help of a veterinary pathologist.

### Statistics

Each of the tests was done in three times and the results are expressed as average value ± SEM.Students t test are used for the statistical analysis of data. A value of *P* < 0.05 was considered as significant.

## Results and Discussion

### Preformulation studies

#### Organoleptic evaluation

The organoleptic character of the drug alpha phellandrene was studied and is shown in Table 2.

### Partition coefficient of the drug

The partition coefficient (log p) of the alpha phellandrene is determined by using oil in water system and is found to be 4.408, indicating that the drug is practically immiscible in water.

### Boiling point of drug

The boiling point of the drug is found to be 171.00°C and is in comply with the monograph (171.00–173.00°C).

### Absorption maxima of the drug

**Figure 1:**
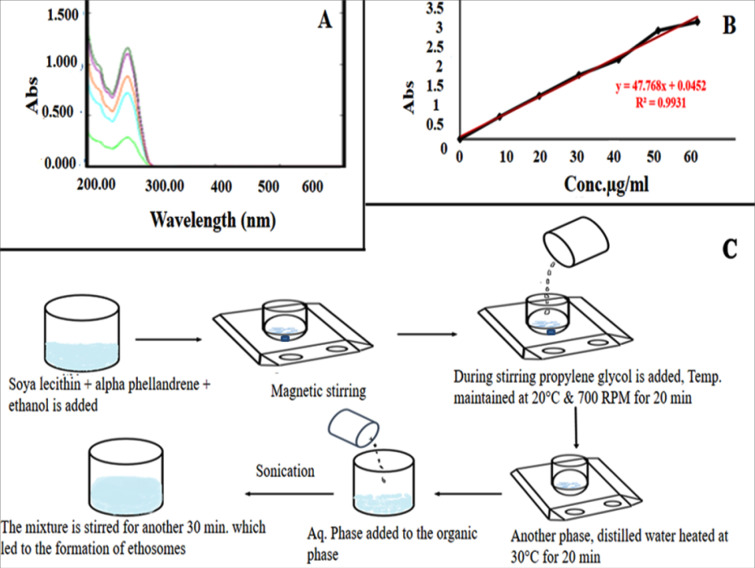
A) Absorption maxima of alpha phellandrene, B) Calibration curve of Alpha phellandrene and C) Schematic representation of preparation of ethosomal suspension

The absorption maximum (λmax) of the drug is found to be 263 nm and it is found to be in accordance with the official standard. The λmax of drug shown in Figure 1A.

### Standard graph of alpha phellandrene

The absorption maximum wavelength of alpha phellandrene is observed to be as 263 nm and the calibration curve (Figure 1B) is constructed by plotting the concentration (X axis) and the absorbance (Y-axis) and the regression coefficient is calculated as 0.99.

### Miscibility studies

The miscibility study of alpha phellandrene in different solvents(Table 3) were carried out and it is found to be miscible with ethanol, dipropylene glycol & paraffin oil and this found to be immiscible with water.

**Table 3:** Comparison of miscibility profile of pure drug with reference

**Solvent**	**Miscibility**	
	**Reference**	**Observation**
Distilled water	Immiscible	Immiscible
Acetone	Immiscible	Immiscible
Ethanol	Miscible	Miscible
Ether	Immiscible	Immiscible
Dipropylene glycol	Miscible	Miscible
Paraffin oil	Miscible	Miscible
Phosphate buffer pH 7.4	Miscible	Miscible

### Formulation studies

#### Preparation of alpha phellandrene loaded ethosomal suspension (APES) and ethosomal gel (APEG)

In the current study APES is formulated by cold method (Figure 1C) and the composition of APES is shown in Table 4. Optimization of formulation is done by varying the concentration of surfactants and co-surfactants.

**Table 4:** Composition of APES

**Components**	**Ingredients**	**Quantity (mg or mL)**
Drug	Alpha phellandrene	42.30
Emulsifier/lubricant	Soya lecithin	5
Solubilizer	Propylene glycol	25
Stabilizer	Cholesterol	2
Co-surfactant	Ethanol	50
Solvent	Distilled water	25

**Figure 2:**
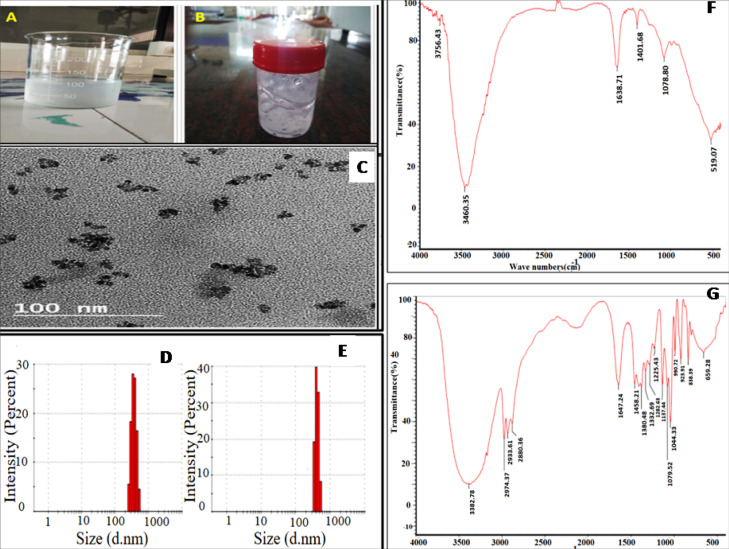
A) Photographs of final formulation of A) alpha phellandrene loaded ethosomal formulation (APES), B) APES loaded carbopol gel (APEG), C) TEM image of APES, D) Particle size of unloaded ethosomal suspension, E) Particle size of APES, F) FT-IR of Alpha phellandrene and G) APES.

Cold method is one of the simplest and most widely used methods for the preparation of ethosomal systems, and if equipped it can be done under nitrogen protection. Technique involves in the preparation of organic phase and aqueous phase separately. The organic phase is obtained by dissolving the phospholipids (in addition to surfactants or penetration enhancer for transethosomes) in ethanol. The aqueous phase used as water, buffer solution or normal saline solution. The method was considered as to be relatively simple without the involvement of complex experimental procedure. The photographs of final formulation are shown in Figure 2A.

Propylene glycol is commonly used as a penetration enhancer. It is used as in the preparation of binary ethosomes at a concentration range of 5%-20% and found to influence the ethosomal properties such as particle size, entrapment efficiency, permeability and stability.^29^ Ethanol is an important component of the ethosomal system. Ethanol plays an important role by giving the unique vesicles characteristics on the terms of particle size, zeta-potential, stability, entrapment efficiency and the skin permeability. Ethanol concentrations in ethosomal systems have been reported to approximately 10%–50%. Reports indicated that when the ethanol concentration is increased, the particle size is decreased. Bendas and Tadros reported that the diameter of ethosomal formulation containing 40% ethanol is 44.6% smaller than that of classical liposomal formulation, which does not, contains ethanol.^30-32^ It is reported that the ethanol modifies the net charge of the systems; provide some degree of steric stabilization, which could be lead to the decrease in mean vesicle size.^33^ However, increasing the ethanol concentration beyond to optimum level would cause the bilayer to become leaky and then lead to slight increase the vesicular size with severe decrease in entrapment efficiency.^34,35^ A further increase in ethanol concentration causes solubilization of vesicles. The selection of the phospholipid type and their concentration are the important factors need to be considered, as it will influences the size, entrapment efficiency, ζ-potential and penetration properties of the vesicles. The different types of phospholipids used in the preparation of ethosomal systems are phospholipon 90G, phospholipon 90H, phospholipon 80H, lipoids S100, lipoids S75-3, lipoids S75, lipoid E80, soya lecithin etc. In general, the concentration of phospholipids in an ethosomal formulation is found to be a 0.5%–5%.^36^ Lopez-Pinto et al found that the incorporation of cholesterol in an ethosomal formulation increases the vesicular stability and rigidity.^37^ For the convenience of topical application we loaded APES into carbopol gel (APEG) and the composition is shown in Table 5.

**Table 5:** Composition of APEG

**Components**	**Ingredients**	**Quantity (mg or mL)**
Polymer	Carbopol	500
Formulation	APES	5
Preservative	Propyl paraben	0.01
Neutralizing agent	Triethanolamine	0.5
Solubilizer	Propylene glycol	5

### Characterization of APES

#### Entrapment efficiency

Entrapment efficiency gives the knowledge about the percentage of alpha phellandrene that is successfully entrapped into the nanoparticles. The entrapment efficiency of APES is found to be 95.06% ± 2.51. Higher entrapment efficiency observed may be because of the presence of ethanol in formulation. It may be because of the increase in aqueous solubility of alpha phellandrene due to the use of higher concentration of ethanol (due to its co-solvent effect), resulting in the more amount of drug to be accommodated within the aqueous core of the vesicles. However when the concentration of ethanol increased above 30% leakage of alpha phellandrene from fluidized bilayer of vesicles, may decrease the entrapment efficiency.^38^ Increasing phospholipid concentration will increase entrapment efficiency significantly. However, the relationship is true only until a certain concentration (0.5%-5%) there after further increase in phospholipid concentration may have no effect on entrapment efficiency. Reported works suggested that the presence of ethanol and propylene glycol in ethosomes provides better solubility of drugs; resulting in higher entrapment efficiency and improved the drug distribution throughout vesicle.

### Particle size & zeta potential

The particle size & zeta potential of formulation is determined out by DLS method. The unloaded ethosomal suspension and APES shows an average particle size of 338.8 ± 29.27 nm (0.412 ± 0.13 PDI) and 364.83 ± 45.84 nm (0.375 ± 0.11 PDI) respectively. The particle size of unloaded ethosomal formulation & APES are shown in Figure 2D & E respectively. The increase in particle size of APES in comparison with unloaded ethosomal formulation may be because of the effective drug lading into the formulation. The particle size is found to be reduced with increase in concentration of surfactant and co-surfactant and the vesicle size is increased with increasing phospholipid concentration; as shown in Table 6.

**Table 6:** Change in particle size with phospholipids of formulation

**S. No.**	**Drug** **(mg)**	**Cholesterol** **(mg)**	**Soya lecithin** **(mg)**	**Ethanol** **(ml)**	**Size ( d.nm )**	**PDI**
F1	42.3	2	5	50	363.83	0.375
F2	42.3	2	10	50	381.60	0.514
F3	42.3	2	15	50	408.80	0.129
F4	42.3	2	20	50	961.70	0.880

Particle size has a major part in both lymphatic uptake and particle retention in lymph node. Particle size range between 10-100 nm is reported to be optimum for lymphatic uptake via subcutaneous administration. Particles having size less than 10 nm is reported to be absorbed via systemic circulation, where as a particles having size larger than 100 nm are preferentially taken up by lymphatic system but at a slower rate. Cholesterol is a steroid molecule and is incorporated in ethosomal systems to enhance its stability and entrapment efficiency of drugs. It prevents leakage, by reducing the vesicular permeability and fusion.^39^ Generally, it is used at a concentration of approximately 3%; but in some cases of formulations it is used up to 70% of the total phospholipid concentration in the formulation. Some studies reported that cholesterol increases the vesicular size of ethosomal systems.^40,41^ Incorporation of propylene glycol in ethosomal systems will leading to the reduction in particle size when compared with the systems without propylene glycol.

Zeta potential measurement is a technique for determining the surface charge of the preparation and it governs the stability of the formulation. The zeta potential of bare ethosomal & APES is found to be -13.3 ± 0.21mV& -19.8 ± 1.27 mV respectively. That means both the formulations exhibited negative surface charge with an increase in zeta value in case of drug loaded formulation. In many cases a *change in zeta potential*  is an indicator of the extent of *drug loading*  in the formulation. However here an increase in zeta potential after drug loading may contribute an increased overall stability of the formulation. In vesicular delivery systems, the charge influences the vesicular properties, such as stability and vesicle–skin interaction. Increase in concentration level of ethanol in ethosomes has shifted the vesicular charge from positive to negative.^42,43^ Dayan and Touitou reported that negative charge of the empty ethosomes is increased with increase in ethanol concentration.^44^ The surfaces of ethosomes acts as a negative charge provider due to the presence of ethanol; causes the electrostatic repulsion between the vesicles and thereby avoid the aggregation of the vesicles. Additionally, ethanol is also reported to have stabilizing effects.^45,46^

### Transmission electron microscopy (TEM)

TEM analysis is used to confirm the shape and size of ethosomal suspension. The prepared APES appeared as multilamellar vesicles with homogenous size below 100 nm (Figure 2C). The ethosome size observed by TEM is appeared as smaller than that of particle size determined by DLS, which may be due to the dehydration & collapse of the hydrophilic corona from the vesicles throughout drying & staining of the TEM specimen.

### Infrared spectroscopic analysis

The FTIR spectra of optimized alpha phellandrene and alpha phellandrene loaded ethosomal suspension formulation are shown in Figure 2F & G respectively. FTIR of alpha phellandrene is characterized by peaks at 3460.35 cm^-1^ (OH group), 1078 cm^-1^ (CO stretch), 1638.71 cm^-1^ (C=O stretch) and at 3756.43 cm^-1^ (Amide group). APES is exhibiting peaks at 3382 cm^-1^ (O-H broad peak), 1458.21 cm^-1^ (Aromatic C=O stretch) and at 2974.37 cm^-1^ (Alkene C-H stretch); broadening of –OH peak along with peak shift may be due to the interaction of drug with excipients in APES.

### Haemocompatibility studies

**Figure 3:**
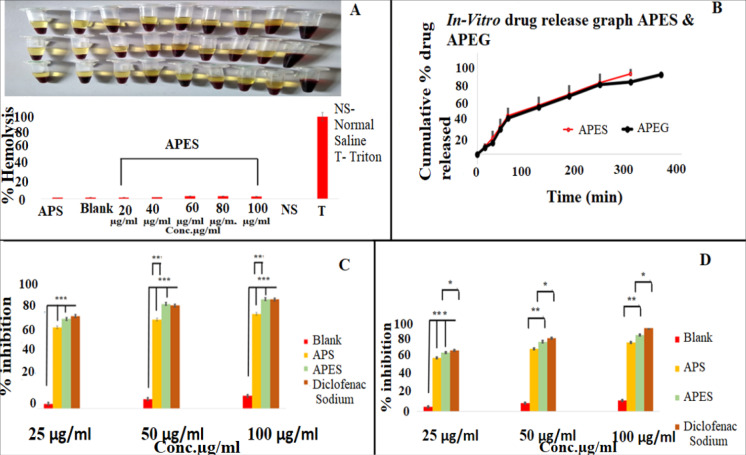
A) *In vitro* hemolysis assay result, B) drug release data, C) COX II activity & D) LOX-5 activity of alpha phellandrene loaded ethosomal suspension (APES).

The haemocompatibility test is carried out to examine the compatibility for the formulation with blood, as they come in contact with the blood on *in vivo* use. The degree of hemolysis is a sensitive indicator of the extent of damage to RBC. In the present study, different concentrations of APES are tested for hemolysis by treating with human blood & incubated for specified period of time. The result indicated that APES not causes any significant hemolysis at all the tested concentration levels as shown in Figure 3A. The % hemolysis observed is less than 5%; which is the recommended standard for a haemocompatible system. It is suggested that the overall –ve charge for the system will prevent its interaction with the –vely charged RBCs and make the system haemocompatible.^31,32^

### In vitro drug release studies

*In vitro* release of alpha phellandrene from APES is evaluated using the dialysis membrane method. Cumulative drug released vs. time graph is plotted. APES & APEG exhibited a faster drug release profile with 95% (after 5 hours) and 94.21% (after 6 hours) of drug get released respectively (Figure 3B). Faster drug release indicates the diffusion of alpha phellandrene from vesicles to the surrounding medium followed by its passage through the cellophane membrane. Higher amount for the ethanol increases in the vehicle movement freely through the membrane and increases the fluidity of ethosomal vesicle bilayers result as increase in the vesicle membrane absorptivity contribute delivery of drug through cellophane membrane.^47^ Here the lipid vesicles are expected to act as a reservoir of drug and provide a continuous delivery of drug.

The *in-vitro* drug release data is subjected to kinetic data analysis by fitting into the data zero order, first order, Korsmeyer-Peppas and Higuchi model. The best fitted release data of APES& APEG is selected based on correlation coefficient values obtained from the selected models. The results suggested that the release of APES follows first order kinetic model with an R^2^ value of 0.9568 & APEG can be best fitted into zero order kinetic model with an R^2^ value of 0.9099. Upon analyzing the release data with Korsmeyer-Peppas equation, the value of release exponent ‘n’ is found to be as1.7343 & 0.0007 for APES & APEG respectively. It is known that for an n value lesser than or equal to 0.45 the system follows Fickian diffusion, n value between 0.45-0.89 non Fickian diffusion, n value greater than or equal to 0.89 super case I which follows diffusion and n value greater than 1 super case II which follows diffusion and erosion as drug release mechanism. The release mechanism for APEG is Fickian diffusion while APES is super case II. Overall kinetic data revealed that APES follows the first order release kinetics with mechanism are drug release as diffusion of erosion.^31,32^

### Cell line studies

RAW 264.7 cell lines are used for the measurements of COX II, 5-LOX, MPO, INOS and cellular nitrite level for assessing the anti-inflammatory activity by in vitro methods.

### Cyclooxygenase II (COX II) activity

Arachidonic acid and their metabolic products; leukotrienes and prostaglandins have been associated with many inflammatory conditions including gout. The whole metabolic pathway of arachidonic acid is regulated by COX-II and lipoxygenase (LOX-5) and augmented activity of these enzymes are associated with inflammation. So compounds that inhibit the activities of COX II and LOX-5 are important for the treatment of inflammatory diseases like gout.^48,49^

COX II is involved in prostaglandin synthesis which is one of the major inflammatory mediators reported in various inflammatory disorders including gout. The inhibiting the activity of enzyme COX II is one of the reported mechanisms of action of currently using anti-inflammatory agents such as NSAIDs. The *in-vitro* anti-inflammatory studies showed that treatment with APES causes the dose dependent inhibition in activity of COX II enzyme and is more/ less comparable with standard drug diclofenac (Figure 3C). Alfa phellandrene solution (APS) & APES at 25µg/mL concentration levels showed 63.2 ± 0.36% and 69.81 ± 4.81 % inhibition in COX II activity respectively. The standard drug diclofenac at the same concentration exhibited a percent inhibitory activity of 72.021 ± 0.004%. At 50 µg/mL concentration percentage inhibition observed with APS, APES and diclofenac are 69.33 ± 0.99, 81.49 ± 5.23 and 80.19 ± 0.10% respectively. Treatment with highest concentration of 100µg/mL; there is 73.75 ± 0.83, 85.06 ± 1.84 and 84.91 ± 0.06% inhibition in COX II activity on treatment with APS, APES and diclofenac respectively. The IC_50_ values of alpha phellandrene from drug solution and APES in COX II inhibition is shown in Table 7.

**Table 7:** IC_50_ values of COX II, LOX-5, MPO& INOS

**Samples**	**IC**_50_ **Values( μg /ml)**			
	**COX II**	**LOX-5**	**MPO**	**INOS**
Alpha phellandrene solution	26.93	37.15	41.666	44.501
APES	28.416	37.31	41.333	42.761
Diclofenac sodium	28.46	39.890	42	42.73

The unloaded ethosome control causes some degree of inhibition in activity of COX-II at the tested concentration levels; it may be because of the anti-inflammatory activity contributed by the components to the formulation. COX-II inhibitory potential of APES at all the tested concentrations are higher than that observed with APS; may be because of the additive effect achieved with blank polymer and alpha phellandrene solution. APES at 50 µg/mL and 100 µg/mL concentrations exhibited COX II inhibitory activity which is little bit higher than that observed with standard drug diclofenac. This expressed that loading into an ethosomal gel did not hindered the anti-inflammatory activity in the selected drug and revealed the suitability of use of the formulation in inflammatory disorder like gout.^23,24^

### Lipoxygenase-5 (LOX 5)

The mechanism of LOX-5 activation is more complex when compared with other lipoxygenases and LOX-5, is responsible for the production of leukotriene; is one of the important mediator of inflammatory reactions. The leukotriene are produced mainly by neutrophils, which are the most abundant mediators reported to be involved in activation of NF-Kβ. NF-Kβ is activated in early stage of joint inflammation and NF-Kβ gene activity is reported to be over expressed in gout patients.^50,51^ So agents that inhibit these enzymes are expected to have anti-inflammatory activity.

APS at 25, 50 and 100 µg/mL showed 62.9 ± 0.07, 73.68± 0.02 and 81.23± 0.01% inhibition in LOX-5 activity respectively. The APES showed an inhibitory activity of 69.32 ± 0.02, 82.23 ± 0.015, 89.84 ± 0.04% at 25 µg/mL, 50 µg/mL and 100 µg/mL concentrations respectively. The standard drug diclofenac sodium at different concentrations of 25 µg/mL, 50 µg/mL and 100 µg/mL produced an inhibitory effect of 71.93 ± 0.93, 86.56 ± 0.93, and 98.02 ± 0.23% respectively (Figure 3B). The % inhibition observed with unloaded ethosome at the highest concentration tested is 12.9 ± 0.132% and which may be because of the anti-inflammatory activity contributed by the components of the formulation. Here also the LOX-5 inhibitory activity of APES at all the tested concentrations are higher than that observed with APS; may be because of the influence of polymer composition and alpha phellandrene solution.

The results of LOX-5inhibitory activity revealed that the treatment with APES causes’ dose dependent inhibition in the activity of LOX-5 and which is more/less comparable with standard drug diclofenac sodium. Comparable anti-inflammatory effect of the formulation with respect to diclofenac sodium suggests the potential use of the formulation in inflammatory conditions like gout.

### Myeloperoxidase (MPO) activity

Myeloperoxidase is an enzyme which is stored in immune cells such as neutrophils and monocytes. During the acute inflammatory reaction activated, neutrophils and monocytes introduce MPO into the injured tissue. MPO has an oxidative and inflammatory effects; it is also reported that non-steroidal anti-inflammatory drugs inhibits MPO activity. MPO is almost abundantly released by neutrophils during gouty attack. The significant correlation between the concentrations of plasma urate and MPO activity is established. The protein NALP3 inflammasome are activated by MSU crystals, which promotes invasion of neutrophils on the site of crystal deposition, which stimulate neutrophils to produce superoxide & other granular proteins, including MPO. Inhibition of MPO activity & leukocyte influx to the affected area may be an indirect indication of the anti-inflammatory activity of the compounds.^52,53^

**Figure 4:**
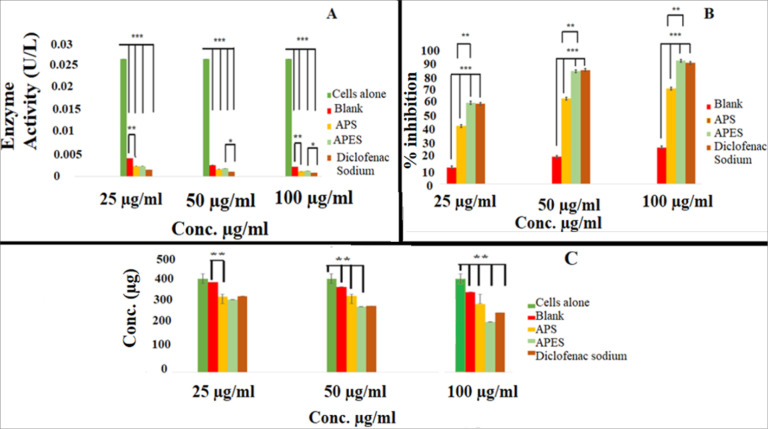
A) Assay results of Myeloperoxidase (MPO) activity, B) Inducible Nitric Oxide Synthase(iNOS) activity and C) Cellular nitrite levels of alpha phellandrene loaded ethosomal suspension (APES) in comparison with alfa phellandrene solution (APS).

RAW cell lines upon induction by APES exhibited an MPO activity of 0.026. The MPO enzyme activity observed with unloaded ethosome control are 0.003972, 0.00247 and 0.002115 U/mL at 25, 50 and 100 µg/mL concentrations levels respectively. MPO activity observed with samples on treatment with APS are 0.00225, 0.00159, and 0.001135 U/mL respectively on treatment with 25, 50, 100 µg/mL concentration levels. APES treated samples showed MPO activities of 0.00228, 0.001797 and 0.001208 U/mL respectively at 25, 50 and 100 µg/mL concentration levels (Figure 4A). The IC50 values for MPO inhibition are shown in Table 7.

Low activity of MPO is an indirect indication of the anti-inflammatory and anti-oxidant properties of the selected drug. Comparable inhibition of activity by APES as that of APS revealed that even after loaded into the ethosomal formulation the drug retains its anti-inflammatory property by inhibiting MPO and is expected to be useful for the treatment of gout. On treatment with standard drug diclofenac sodium MPO activities observed are 0.001419, 0.00099 and 0.000759 U/mL respectively at the tested levels of 25, 50 and 100 µg/mL. Diclofenac is a widely accepted anti-inflammatory agent and hence exhibited greater inhibition in MPO activity than that observed with APES.

### Inducible nitric oxide synthase

The inducible NOS (iNOS), is normally not expressed in resting cells and it has to be induced by certain cytokines or microbial products. At inflammatory conditions, inducible nitric oxide synthase (iNOS or type II NOS) produce nitric oxide, which is reported to be a true inflammatory mediator. A number of inflammatory and infectious diseases are mediated by nitric oxide and it exhibits its action as a direct effectors and as a regulator of other pathways. Agents which inhibit the activity of iNOS are said to posse’s anti-inflammatory activity. The results clearly indicate that at 25 µg/mL concentration the APS, APES and diclofenac exhibited 45±0.01, 60.81±0.01 and 61.58 ± 0.02% inhibition in activity of iNOS. At 50 µg/mL concentration 64.8±0.01, 85.6±0.02% and 86.48 ± 0.01% inhibition in activity of iNOS is caused by APS, APES and standard drug diclofenac sodium respectively. On treatment with 100 µg/mL concentration there is 72.19± 0.01, 93.6 ±0.06 and 91.89±0.03 % and 91.89±0.03% inhibition in iNOS activity is caused by APS, APES and diclofenac treatments respectively (Figure 4B). The IC_50_ values for iNOS inhibition are shown inTable 7.

It is clear that there is dose dependent inhibition in activity of iNOS on treatment with the APES and the activity is very much comparable with that of the standard drug diclofenac. Significant inhibitory activity of inducible form of nitric oxide synthase enzyme is expected to be useful for the treatment of inflammatory disease gout; as iNOS is reported to be augmented in inflammatory conditions. One of the mechanisms of anti-inflammatory effect of alpha phellandrene is reported to be mediated through the reduction of cellular nitric oxide level by decreasing the activity of INOS enzymatic activity.^52-55^

### Evaluation of Cellular Nitrite Levels

Generation of nitrite is the end result of activity of iNOS. RAW cell lines upon induction by LPS caused cellular nitrite level of 416.79 µg. Quantification of cellular nitrite level on treatment with various samples revealed that treatment with diclofenac sodium at 25, 50 and 100 µg/mL treatment causes a generation of 339 ± 3, 295±1.12 and 265.36±4µg nitrite respectively. APES treatment at 25, 50 and 100 µg/mL causes a net generation of 324±6.08, 292±2 and 224 ±4 µg of nitrite respectively (Figure 4C). Low level of nitrite generation may be due to the suppressed activity of iNOS caused by the formulation treatments and represent the anti-inflammatory property of the formulation. The effect of alpha phellandrene on cellular nitrite level may be because of its terpenes nature as s terpenoids are reported as inhibitors of free radical or scavenger; possibly act by oxidizing the radicals and there by inhibit inflammation. Treatment with APS causes a net nitrite generation of 336±4.3, 340±2 and 303±2 µg at 25 µg/mL, 50 µg/mL & 100 µg/mL of concentrations respectively. The unloaded ethosome formulation showed the nitrite level of 401±1.73, 380±0.57 and 357±1.52 µg at 25, 50 and 100 µg/mL concentration respectively.

### In vitro skin permeation study

**Figure 5:**
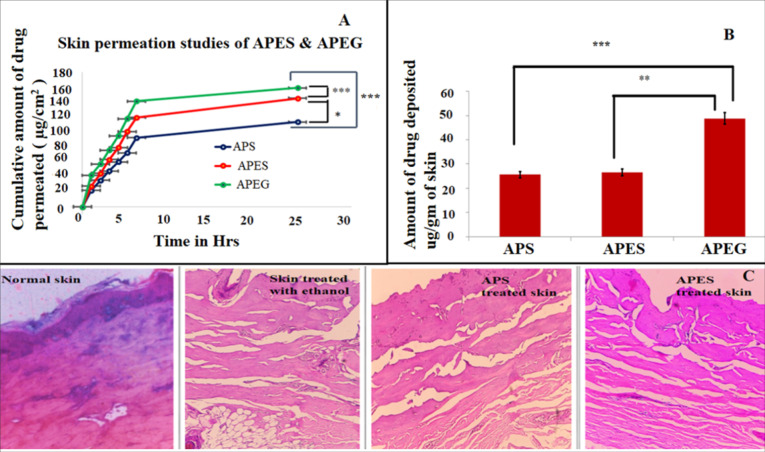
A) Skin permeation data, B) Skin deposition data and C) Skin histology after various treatments

The *in vitro* skin permeation study is carried out using the solution of APES & APEG in ethanol. The total amount of drug passed through the skin vs. time graph is plotted and shown in Figure 5A.

The amount of drug permeated from APES & APEG is found to be 159.00 ± 19.28µg/cm^2^& 145.13 ± 14.16 µg/cm^2^ after 24 hours respectively. The steady state flux, enhancement ratio & permeation constant are shown in Table 8.

**Table 8:** Steady state flux, enhancement ratio & permeation coefficient of alpha phellandrene based on skin permeation studies

**Sample**	**Steady state flux (µg/cm2/h)**	**Enhancement ratio**	**Permeation coefficient (cm2/h)**
Alpha phellandrene solution	3.905	-	0.0039
APEG	4.9856	1.276	0.0049
APES	5.184	1.3275	0.0051

Numerous studies reported the improved skin penetration of xenobiotics through the ethosomal formulations. Ethosomes; like classical liposomes not only contain phospholipids but also have excessive levels of ethanol; will able for enhance the penetration to deep tissues into the systemic circulation. Ethanol interacts with lipid molecules through its polar head group region, reduces the phase transition temperature of the stratum corneum lipids and there by increases the fluidity which leads to improved membrane permeability. It further contributes softness and flexibility; that allows ethosomes to squeeze very easily and penetrate into the deeper layers of the skin. Reduction in particle size to nano may also contribute enhanced permeation of ethosomal formulation. Ethosomal gel preparations are reported to be much more effective permeation enhancers than hydroethanolic solution, ethanol and ethanolic phospholipid solution. Some authors are reported that same superior properties in ethosomal gels for different drugs/agents over traditional gels. Interestingly, it is found that drug-release rate from the ethosomal suspension (APES) is faster than that of ethosomal gel (APEG), it may be due to the high viscosity of gel offered by carbopol.^11-14^

### Skin deposition study

The drug is deposited in skin from APEG is significantly higher than that observed with APS & APES treatments (Figure 5B). Amount of alpha phellandrene deposited from APS 25.596 ± 0.32 µg/cm^2^. From APES and APEG amount of alpha phellandrene deposited in skin are 26.844 ± 0.26 µg/cm^2^ and 48.799 ± 1.547 µg/cm^2^ at 24 hours respectively. The amount of alpha phellandrene deposited from APEG is comparatively high, because of the viscosity contributed by carbopol as it is retained in the surface layer of skin. Permeation studies along with drug retention studies revealed that APES provide sufficient permeation to the drug alpha phellandrene without causing much retention when compared with drug solution and expected to provide sufficient anti-gout activity.^12,13^

### Skin histology

Hematoxylin-eosin stained images of skin samples after various treatments are shown in Figure 5C. Normal porcine skin is characterized by the presence of stratum corneum (S.C) , with well-organized layers of epidemic & underlying layer of dermis. Skin treated with ethanol is characterized completely ulcerated surface epidermis with underlying by dermis show ectatic capillaries; and inflammatory infiltrate of neutrophils. Upon visual examination of porcine skin for irritation on APS & APES treatment, no sign of inflammation is observed. Histology revealed mild degree of inflammation in both the cases may be due to the presence of ethanol content in the samples. However no apparent change in dermis region is observed.^12,13^

## Conclusion

Gout is an inflammatory arthritis associated with hyperuriceamia, resulting from MSU crystals deposition in joints, tendons and surrounding tissues. Prevalence rate of gout is about 1.4% in common population: increased incidence of gout is reported nowadays; due to poor dietary habits, lack of exercise, obesity & metabolic syndrome. The drugs used for the treatment of acute and chronic gout are NSAIDs, colchicine, steroids, allopurinol, probenecid, sulfinpyrazone and are associated with side effects such as headache, dizziness, nausea, heart, kidney problems etc. The use of plant based drugs for the treatment of gout is increasing now days; because these are much safer, when compared with synthetic drug. *Moringa oleifera*, (family, Moringaceae); the plant has been reported to possess many pharmacological activities and is rich in alkaloids, saponins, terpenes, phenolic acids, steroids, glucosinolates, flavonoids. Alpha phellandrene belongs to the terpenes component of *Moringa* and potentially useful for the treatment of various inflammatory diseases like rheumatoid arthritis, osteoarthritis, allergic disease etc. But the oral use of alpha phellandrene is reported to cause gastric disturbances, vomiting, nausea etc. Skin irritation is reported on topical use of alpha phellandrene. The present study focused towards the development of a topical ethosomal formation of alpha phellandrene. The particle size of APES is found to be suitable for improving the permeation of the selected drug, and is found to be haemocompatbile. The improved skin permeation with deposition indirectly indicated the effectiveness of the formulation by topical use & is expected to be an alternative for oral use of alpha phellandrene. However further animal studies are essential to reveal the actual anti-gout potential of the formulation on topical application.

## Ethical Issues

Not applicable.

## Conflicts of Interest

The authors have no affiliation or ﬁnancial conflict with the subject of materials discussed in the manuscript with any of the organization or entity.
